# Extracellular vesicle signatures are modified in haemolymph of atlantic rock crab (*Cancer irroratus*) infected with shell disease – a Study from Icelandic waters

**DOI:** 10.1016/j.cirep.2026.200299

**Published:** 2026-07-09

**Authors:** Sigrun Lange, Sindri Gíslason, Sarah R. Needham, Benjamin M. Davis, Igor Kraev, Hermann Dreki Guls, Sandra Dögg Georgsdóttir, Árni Kristmundsson, Halldór Pálmar Halldórsson

**Affiliations:** a Pathobiology and Extracellular Vesicles Research Group, School of Life Sciences, University of Westminster, 115 New Cavendish Street, London, W1W 6UW, UK; b Southwest Iceland Nature Research Centre (SINRC), Garðvegur 1, 245, Suðurnesjabær, Iceland; c Central Laser Facility, UKRI: Science & Technology Facilities Council, Rutherford Appleton Laboratory, Oxfordshire, UK; d Electron Microscopy Suite, Faculty of Science, Technology, Engineering and Mathematics, The Open University, Milton Keynes, UK; e The University of Iceland´s Research Centre in Suðurnes (UIRCS), University of Iceland, Garðvegur 1, 245, Suðurnesjabær, Iceland; f Fish Disease Laboratory, Institute of Experimental Pathology, Keldur, University of Iceland, 112, Reykjavík, Iceland

**Keywords:** Cancer irroratus, DSTORM, Extracellular vesicles, Infection, Shell disease, Proteomics, Gene ontology, Cell-communication, Immunity, Biomarker

## Abstract

Crustacean shell disease is characterised by degradation of the chitinous exoskeleton, with subsequent microbial invasion and septicaemic infections, which can be exacerbated by environmental stressors including temperature fluctuations and pollutants. Recent research on shell disease in Atlantic rock crab (*Cancer irroratus*) in Icelandic waters has raised significant concern of potential impacts of this non-indigenous species on the native ecosystem. Increased understanding of cell communication mechanisms associated with shell disease is therefore important. Extracellular vesicles (EVs) play critical roles in cellular communication by cell-cell transfer of various cargo species and can be isolated from biofluids. To investigate systemic responses with respect to altered EV communication in *C. irroratus* with shell disease, this study assessed haemolymph EV profiles, including proteomic content, from infected and non-infected male and female crabs. Gene ontology and Reactome pathway enrichment analysis associated crab haemolymph EV protein cargoes with a range of immune, cell communication, membrane trafficking, vesicle-mediated transport cell cycle, signal transduction, transcriptional regulation, as well as DNA damage and repair response pathways. Overall, 34 proteins were identified with some sex-differences observed. Two proteins only identified in EV cargoes of infected male crabs were alpha-2-Macroglobulin, which has key innate immune functions, and actin-1 which has multifaceted roles in immunity and cell communication. Three proteins identified only in EV protein cargoes of the infected female crabs were Enkurin domain containing protein, which as roles in the cell cycle and DNA repair; ribosomal protein Lu5, which has roles in translation, metabolism and RNA processing; and Discoidal lipoprotein and beta-glucan binding protein with unidentified roles. Concentration of EVs in haemolymph was elevated in the male crabs with shell disease, compared with non-infected individuals and some changes were observed in EV subpopulation dynamics between male and female crabs in response to infection, based on nanoparticle tracking analysis and single-molecule localisation-based super-resolution microscopy imaging of crab haemolymph EVs. The findings pave way for using haemolymph EVs as biomarkers for monitoring disease responses in marine shellfish, with potential for translatability for studying disease epidemics in invasive marine species globally.

## Highlights

• Extracellular vesicle signatures were profiled in *C. irroratus* by NTA, proteomics and dSTORM.
• Haemolymph EV subpopulation dynamics were modified in male and female crabs with shell disease.
• Haemolymph EV proteome cargoes associated with immune, cell communication and cell cycle pathways.
• Haemolymph EV proteomes showed some sex specific differences in shell disease.
• Alpha-2-Macroglobulin and actin-1 were specific to infected male EVs.
• Enkurin, Ribosomal protein Lu5 and Discoidal lipoprotein were specific to infected female EVs.

## Introduction

Crustacean shell disease has been reported worldwide in various species and is characterised by degradation of the chitinous exoskeleton. The disease has multifaceted causes, comprised of initial insult due to natural behaviours such as fighting, predation, cannibalism and abrasion against sediment and surfaces [1,2]. Additional environmental stressors include pollutants and temperature fluctuations [[3], [4], [5]]. Furthermore, progressive shell degradation occurs due to various chitinolytic microorganisms, and dysbiosis of different chitinolytic and lipolytic bacteria [[6], [7], [8], [9]]. This can lead to subsequent microbial invasion (bacterial, fungal) and septicaemic infections [10,11], in addition to unsuccessful moulting [12].

Recent research on population dynamics and shell disease in Atlantic rock crab (*Cancer irroratus*) in Icelandic waters has raised significant concern of potential impacts of this non-indigenous species on other shellfish species and the native ecosystem [[13], [14], [15]]. A recent in-depth histopathological study of the shell disease in *C. irroratus* revealed synergistic effects of various bacterial and oomycete pathogens [15]. Therefore, increased understanding of cell communication mechanisms associated with shell disease in *C. irroratus* is of considerable interest for biomarker discovery and may furthermore have potential translatability for studying disease epidemics in invasive marine species globally.

Extracellular vesicles (EVs) are small membrane bound vesicles in the range of 40–500 nm, released from and taken up by cells, as part of cellular communication. EVs play multifaceted roles in physiological and pathobiological processes and can be isolated from most biofluids. As EVs carry a range of cargoes including RNA species, DNA, lipids and proteins, they are widely studied as biomarkers in human pathologies [16,17]. EV mediated cell communication is a phylogenetically conserved mechanism [[18], [19]], and there is an increasing interest in the use of EVs as indicative biomarkers for health and disease monitoring in aquatic species [[20], [21], [22], [23], [24], [25], [26], [27], [28], [29], [30]]. Recent publications on crustaceans have shown that EVs can be isolated from haemolymph and provide indication of cellular processes and immune responses [20,[31], [32], [33], [34], [35], [36]].

To investigate systemic responses with respect to altered EV communication in *C. irroratus* with shell disease, this study assessed haemolymph EV profiles, including proteomic content, from infected and non-infected male and female crabs. We hypothesize that EV signatures are modified in the haemolymph of crabs with shell disease, including possible sex specific differences.

## Materials and methods

### Sampling and experimental setup

**Fig. 1:**
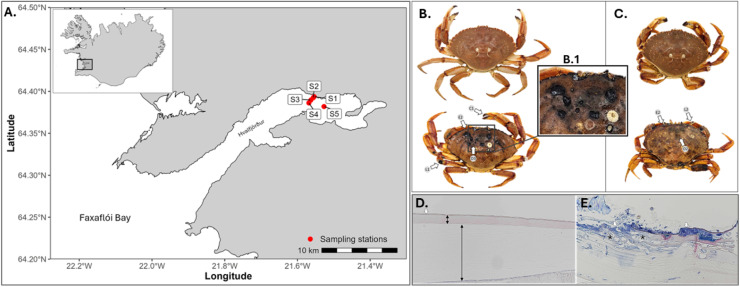
Sampling location of crabs used in the study and representative examples of shell disease. A. Sampling location and the five sampling stations (S1-S5) are highlighted (red circles) in Hvalfjörður, SW Iceland. **B.** Representative images of healthy and infected male crabs; inset shows a close-up of lesions on the carapace (B.1). **C.** Representative images of healthy and infected female crabs. The distribution of lesions across the exoskeleton of infected individuals is indicated by arrows and numerical labels corresponding to the infection severity scale described in [15]. **d-E.** Giemsa-stained histological sections. **D.** Normal structure of the exoskeleton of *C. irroratus*, showing the outermost non-chitinous epicuticle (white arrow) and the underlying chitin-containing exo- and endocuticle (black arrows). **E.** Severely eroded and necrotic cuticular layers (asterisk *), associated with masses of bacteria (white arrows).

Crabs were sampled by trap fishing from five sampling stations (S1-S5; Fig. 1A) in Hvalfjörður, a 35 km long and 3.5 km wide fjord with maximum depth of 84 m, in SW-Iceland, as previously described in [15]. *C. irroratus* was captured using commercial crab traps (©GuangJin Group Ltd., height 30 cm, length 80 cm, width 40 cm, mesh size 4.8 cm, with the escape opening for juveniles closed). Traps were baited with a mixture of fish, including gadoids (*Gadus morhua, Pollachius virens, Melanogrammus aeglefinus*). Mixed bait was placed in mesh bags hanging in the traps, ca. 500 g per trap. Baited traps remained in place for about 48 h before retrieval. Captured crabs were identified by size (carapace width (CW) was measured to the nearest 0.1 cm between the two most distant points on the carapace, using a vernier caliper) and sex, and shell disease was assessed by visible shell lesions according to specific locations on the crab exoskeleton (claws, dorsal carapace, carapace edge, legs) according to previously set parameters in Gíslason et al. [15], and are summarised for the individual crabs in this study in Supplementary Table 1. with scoring criteria based on the distribution and numerical intensity of exoskeleton lesions/spots. Crabs were categorized as infected if they exhibited lesions in any of the following categories: Claws (C1: initial signs, C2: focal spots, C3: broken tips); Dorsal carapace (D1: 1–5 spots, D2: 6–10 spots, D3: >10 spots/all over); Edge of carapace (E1: <40% coverage, E2: >40% coverage) or Legs (L1: <5 spots, L2: >5 spots. Crabs categorized as non-infected (control) showed a total absence of visible exoskeleton lesions receiving a score of zero across all categories.Fig. 1 shows representative images of infected and non-infected male (Fig. 1B) and female crabs (Fig. 1C) respectively, with arrows pointing to the lesions and are further highlighted in the magnified image provided (Fig. 1, B.1). The histopathology of the shell disease has previously been described in detail [15], and representative Giemsa-stained histological sections are shown in Fig. 1d-E, with normal non-infected structure of the exoskeleton presented (Fig. 1D), compared with severely eroded and necrotic cuticular layers associated with masses of bacteria in the infected shell (Fig. 1E).

Following capture, crabs were visually assessed and categorized as infected or non-infected on board the research vessel (See supplementary Table S1). To minimize physiological variability, only inter-moult (hard shell) crabs were included in the study to avoid significant haemolymph protein fluctuations that may be associated with the moulting cycle. Body size (carapace width) was controlled by selecting individuals within a narrow size range for each sex as body size and inter-moult duration can influence shell disease dynamics [15]. Furthermore, while crabs were captured from five localized stations in Hvalfjörður, no significant differences in infection characteristics were observed between stations and individuals were pooled by infection status (infected versus non-infected) and sex (male/female) for subsequent EV analysis. The crabs were transported live to the UIRCS research facility in Sandgerði, Iceland, where they were kept for five days in separate tanks per group supplied with continuous flowing borehole seawater (approximately 9.5 °C) and not fed until haemolymph samples were collected. This standardised fasting period was implemented to eliminate fluctuations in haemolymph metabolism, lipid levels and protein concentrations which could possibly interfere with EV signatures. Haemolymph was collected from each crab (as more males were caught in this expedition samples were collected from n = 20 of infected and non-infected males; but n = 10 of infected and non-infected females, respectively), by inserting a 22-gauge hypodermic needle attached to a sterile syringe through the arthrodial membrane of a walking leg, collecting 200- 500 uL aliquots per individual. Samples were stored at −80 °C until EV processing.

**Fig. 2:**
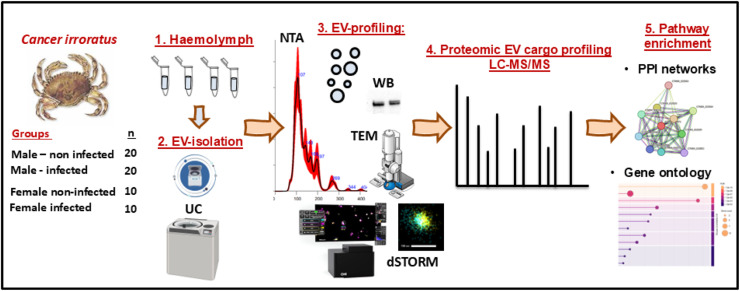
Experimental workflow. Atlantic rock crab (*C.irroratus*) haemolymph was collected from non-infected and infected male and female crabs respectively. EVs were isolated by differential centrifugation and ultracentrifugation (UC) with EV profiling carried out by nanoparticle tracking analysis (NTA), western blotting (WB), transmission electron microscopy (TEM) and direct stochastic optical reconstruction Microscopy (dSTORM). EV protein cargoes were identified by LC-MS/MS and assessed for protein-protein interaction networks (PPI) and associated gene ontology pathway enrichment.

The experimental setup and workflow are summarised in Fig. 2, with haemolymph EV isolation and subsequent EV profiling by nanoparticle tracking analysis (NTA), western blotting (WB) for surface markers, transmission electron microscopy (TEM) and direct stochastic optical reconstruction microscopy (dSTORM) imaging of EVs for morphology and surface marker detection. EV protein cargoes were identified by LC-MS/MS with protein hits analysed for protein-protein interaction (PPI) networks and associated pathway enrichment analysis for Gene ontology (GO) and Reactome pathways.

### EV isolation and characterisation

EVs were isolated from crab haemolymph by differential centrifugation and ultracentrifugation according to methods previously standardised and published from our laboratory, including for crustacean haemolymph samples [20,31]. In brief, haemolymph samples were diluted 1/5 in DPBS (100 µL haemolymph per individual sample plus 400 µL DPBS, filtered in 20 µm filter to avoid any contamination with particles from DPBS) and centrifuged at 4000 × *g* for 20 min at 4 °C, to discard any aggregates. The EV containing supernatants were then moved into fresh Eppendorf tubes and centrifuged at 25,000 × *g* for 1 h at 4 °C using Microtube rotor (75,003,652) at maximum speed in the High Speed Multifuge X1R Centrifuge (ThermoScientific). The resulting total EV enriched pellet was kept, discarding the supernatant, and adding fresh 500 µL of DPBS to wash the enriched EV pellet, followed by a second centrifugation for 1 h at 25,000 × *g*, at 4 °C. The final EV pellets were each resuspended in 100 µL of DPBS before further processing by nanoparticle tracking analysis (NTA), transmission electron microscopy (TEM), dSTORM imaging, SDS-PAGE, Western blotting and LC-MS/MS.

#### Nanoparticle tracking analysis

EVs were quantified, and size profiles were generated using the NS300 Nanosight (Malvern Panalytical, Malvern, UK) for nanoparticle tracking analysis (NTA), using a 488 nm blue laser, with the syringe pump speed set at 50 and the camera level set at 12 for capture. Samples were recorded for 4 × 60 s, and histograms were generated by averaging the four readings per sample; the threshold was set at level 5 for post-analysis processing. The NTA reports generated using the NTA software (version 3, Malvern, UK) were used to identify the quantity of EVs/mL, modal size, and proportion of EV subpopulations based on size: small EVs (sEVs, ≤100 nm), medium-sized EVs (mEVs, 101–200 nm), and large EVs (lEVs, >200 nm).

#### Transmission electron microscopy (TEM)

For imaging preparation of EVs by TEM, EV pellets were resuspended in 100 mM sodium cacodylate buffer (pH 7.4). Approximately 3–5 μL of the EV suspension was applied to a glow-discharged TEM grid with a carbon support film. The sample was partially air-dried for around 10 min before the grid was placed onto a drop of fixative solution (2.5% glutaraldehyde (Agar Scientific Ltd., Stansted, UK) in 100 mM sodium cacodylate buffer, pH 7.4) for 1 min at RT. The grid was subsequently transferred across three drops of distilled water for washing, with excess water being removed using filter paper. Then, the grid was placed onto a drop of staining solution of 2% aqueous uranyl acetate (Agar Scientific Ltd., Stansted, UK) for 1 min, and any excess stain was removed with filter paper before air drying. EVs were imaged using a JEOL JEM 1400 microscope (JEOL, Tokyo, Japan) operated at 80 kV, with magnifications ranging from 30,000 × to 60,000 × . Digital images were captured using an AMT XR60 CCD camera (Deben, Bury Saint Edmunds, UK).

#### Direct stochastic optical reconstruction microscopy (dSTORM) imaging

Single-molecule localisation-based super-resolution microscopy imaging of crab haemolymph EVs was carried out using the ONI EV profiler 2 kit and ONI Nanoimager system (Oxford Nano Imaging, ONI, Oxford, UK). EVs were pooled from 10 individuals per sample group (non-infected and infected males and females, respectively), and EV samples were prepared according to the manufacturer’s instructions (ONI), following the manual sample preparation protocol (EV Profiler 2), using phosphatidylserine (PS) capture for capturing the EVs. EV visualisation was carried out with fluorophore labelled antibodies against EV specific surface markers, comprised of tetraspanin trio (TT; anti-CD9 + CD63 + CD81 (561)) and a pan-EV marker (488), using the AutoEV imaging and CODI analysis protocol according to the manufacturer’s specifications (ONI). For quantification of labelled EVs by dSTORM, the datasets (EV positivity) were recovered using the particle filters: diameter range 50 - 500 nm, with label counts > 5 for EV characterisation markers (pan-EV and TT), from 6 to 12 fields of view per sample group (50 × 80 µm) [37]. EV densities (particles / m^2) for each labelling condition are reported.

#### SDS-PAGE, silver staining and western blotting

Crab haemolymph EV pools (n = 10), for the four experimental groups respectively (infected and non-infected males and females) were used to detect total protein EV content by silver-staining and for EV surface marker detection by western blotting. The EV preparations were diluted in 2 × Laemmli sample buffer (BioRad), boiled at 100 °C for 5 min for denaturation, loaded at approximately 5 μg/lane and separated on 4–20% TGX gels (BioRad) at 165 V for 55 min. Gels were either stained for total protein by silver-staining (BioRad Silver Stain Plus Kit (1610,449, BioRad), following the manufacturer’s protocol), or proteins were transferred to nitrocellulose membranes by semi-dry transfer for Western blotting at 15 V for 1 h. The membranes were blocked in 5% bovine serum albumin (BSA, Sigma-Aldrich) in TBS-T for 1 h at room temperature (RT), followed by primary antibody incubation with CD63 (ab216130, Abcam, Cambridge, UK) and Flotillin-1 (ab41927) overnight at 4 °C on a shaking platform. The membranes were washed 3 × 10 min in TBS-T and incubated in secondary HRP-labelled anti-rabbit IgG (BioRad, diluted 1/3000 in TBS-T) antibody for 1 h at RT. The membranes were washed for 5 × 10 min in TBS-T and developed using enhanced chemiluminescence (ECL, Amersham Biosciences, Buckinghamshire, UK) in conjunction with the UVP BioDoc-ITTM System (Thermo Fisher Scientific, Dartford, UK).

### Liquid chromatography-tandem mass spectrometry (LC-MS/MS) of EV proteome cargoes

Total proteins were isolated from crab haemolymph EV pools, for the four experimental groups respectively (infected and non-infected males and females) with pools from 10 individuals per group. Proteins were extracted using RIPA+ buffer (Sigma-Aldrich, Gillingham, UK, containing 10% protease inhibitor cocktail, Sigma-Aldrich), incubated for 2.5 h on a continuously rotating platform at 4 °C. The samples were centrifuged at 16,000 × *g* for 30 min at 4 °C, and the protein-containing supernatant was collected. Proteins were diluted in 2 × Laemmli sample buffer (BioRad), loaded at 5 μg/ well and run 0.5 cm into a 12% TGX gel (BioRad), and thereafter cut out as one gel band per group for in-gel digestion followed by LC-MS/MS, performed by Cambridge Proteomics (University of Cambridge, Cambridge, UK). In brief, automated LC-MS/MS analysis was carried out using a Dionex Ultimate 3000 RSLC nanoUPLC system (Thermo Fisher Scientific Inc., Waltham, MA, USA) in conjunction with a QExactive Orbitrap mass spectrometer (Thermo Fisher Scientific Inc., Waltham, MA, USA). Separation of peptides was performed by reverse-phase chromatography at a flow rate of 300 nL/min and a Thermo Scientific reverse-phase nano Easy-Spray column (Thermo Scientific PepMap C18, 2 mm particle size, 100A pore size, 75 mm i.d. × 50 cm length). Peptides were loaded onto a pre-column (Thermo Scientific PepMap 100 C18, 5 mm particle size, 100A pore size, 300 mm i.d. × 5 mm length) from the Ultimate 3000 autosampler with 0.1% formic acid for 3 min at a flow rate of 15 mL/min. After this period, the column valve was switched to allow elution of peptides from the pre-column onto the analytical column. Solvent A was water + 0.1% formic acid and solvent B was 80% acetonitrile, 20% water + 0.1% formic acid. The linear gradient employed was 2–40% B in 40 min. Further wash and equilibration steps resulted in a total run time of 60 min. The LC eluent was sprayed into the mass spectrometer using an Easy-Spray source (Thermo Fisher Scientific Inc.). The *m/z* values of all eluting ions were measured in an Orbitrap mass analyser, and data-dependent scans (selecting top 20) were employed for automatic isolation and generation of fragment ions using the HCD collision cell, measured using the Orbitrap analyser (ESI-ORBITRAP-HCD). Both singly charged ions as well as ions with unassigned charge states were excluded from selection for MS/MS. A dynamic exclusion window of 20 s was applied. The data were processed post-run using Protein Discoverer (version 2.1., Thermo Fisher Scientific Inc.), converted to mgf files, and submitted to Mascot (Mascot search algorithm; Matrix Science, London, UK). Searching for hits was carried out against the UniProt database Heterotremata Heterotremata_20,260,203 (173,062 sequences; 44,526,601 residues) with peptide and fragment mass tolerances, respectively, set at 20 ppm and 0.1 Da. The threshold value for significance was set at *p* < 0.05, and the peptide cutoff score was set at 33.

### Protein-protein interaction network and functional pathway enrichment analysis of crab haemolymph EV proteome cargoes

For the construction of protein–protein interaction (PPI) networks and associated pathway enrichment analysis, STRING (Search Tool for the Retrieval of Interacting Genes/Proteins; https://string-db.org/) analysis was applied. The protein networks were built based on the protein names, using the Crustacea STRING database with settings set at medium confidence. Colour lines connecting the nodes represent the following evidence-based interactions for the network edges: “known interactions” (this is based on experimentally determined data or curated databases); “predicted interactions” (this is based on gene co-occurrence, gene neighbourhood or gene fusion); “others” (this is based on co-expression, text mining or protein homology. Networks were assessed for Gene Ontology (Biological function) and Reactome pathways. The *Penaeus vannamei* STRING database had to be used as representative for Crustacea, for the creation of the networks and associated pathway enrichment analysis, as no crab-specific STRING database is available, and as *Penaeus vannamei* showed the most hit number identity with the proteins identified in *C. irroratus* EVs.

### Statistical analysis

For comparison of EV datasets from the four study groups, GraphPad Prism version 10 was used; normal distribution of the data was confirmed by Shapiro-Wilk test. One-way ANOVA or *t*-test were used with statistical significance regarded as *p* < 0.05. STRING analysis was carried out with medium confidence, and the maximum number of interactors was set for the first shell query proteins only (https://string-db.org/, accessed on 23 January 2026).

## Results

### Profile analysis of EVs from haemolymph of infected and non-infected crabs

**Fig. 3:**
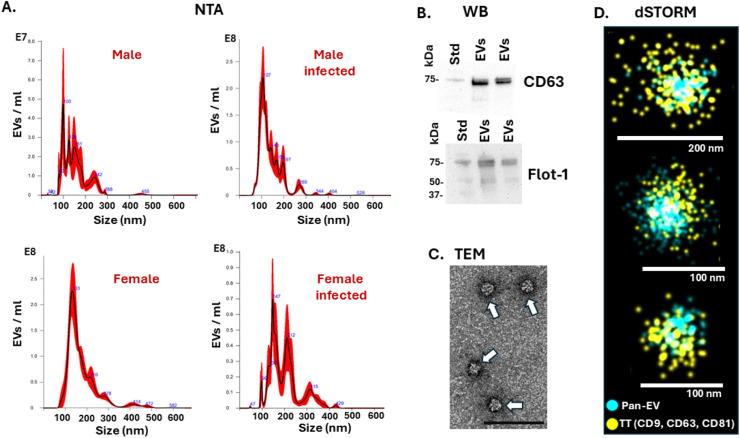
Characterisation of EVs from crab haemolymph. A. NTA analysis showing representative size distribution curves from EVs of the four groups – male non-infected, male infected, female non-infected, female infected; **B.** Western blotting (WB) of crab haemolymph EVs for CD63 and flotillin-1 shows positive detection; **C.**TEM imaging of crab haemolymph EVs (white arrows); **D.** Representative images from dSTORM imaging of crab haemolymph EVs, co-stained with pan-EV marker and tetraspanin-trio EV marker TT (CD9, CD63, CD81).

Crab haemolymph EVs showed size distribution profiles in the range of approximately 40 to 400 nm by nanoparticle tracking analysis (NTA) for all groups, with representative NTA curves shown for each group in Fig. 3A (male non-infected and infected; female non-infected and infected). EVs were assessed for CD63 and Flot-1 by Western blotting (Fig. 3B), imaged by transmission electron microscopy (TEM; Fig. 3C), and by super resolution microscopy for pan-EV and tetraspanin trio markers using dSTORM with the EV2 Profiler kit (Fig. 3D).

**Fig. 4:**
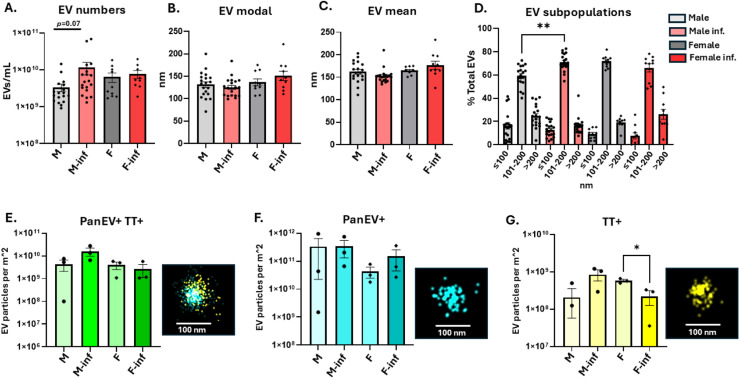
Haemolymph EV profiles of infected and non-infected crabs by NTA (A-D) and dSTORM imaging (E-G). A. EV concentration in haemolymph (EVs/mL), *p ≤ 0.05, ANOVA; **B.** EV modal size (nm); **C.** EV mean size (nm); **D.** EV subpopulation distribution as percentage of total EVs, assessing small EVs (≤100 nm), medium EVs (101–200 nm) and large EVs (>200 nm) **p ≤ 0.01; ANOVA used in A-D; n = 20 for male groups and n = 10 for female groups in A-D. **E-G.** dSTORM labelling comparison for EVs across the four groups, comparing PanEV and TT co-labelled EVs (**E**), PanEV labelled EVs only (**F**) and TT labelled EVs only (**G**); n = 3 replicate readings for ONI chips in 6–12 fields of view for E-G; y-axis represents EV density (EV particles per m^2); p*≤0.05, *t*-test. Error bars represent standard error of mean (SEM).

EVs from haemolymph of all four groups were quantified and profiled by NTA, with respect to EV numbers/mL (Fig. 4A), EV modal size (Fig. 4B), EV mean size (Fig. 4C) and EV sub-populations based on size, with small EVs ≤100 nm; medium EVs 101–200 nm and large EVs >200 nm (Fig. 4D). EV concentration was in the overall range of 1 × 10^9^ to 1 × 10^10^, with some individual variations observed within each group, and showed a trend for elevation in the infected males (*p* = 0.07, ANOVA), while modal size (ranging from 60–200 nm, with individual variations within each group) and mean size (overall in the 100–200 nm range, with individual variations within each group) were not significantly changed between the four study groups. When assessing the distribution of EV subpopulations based on size by NTA analysis, the majority of total EVs for all four groups fell within the medium EV size range at 101–200 nm, with a statistically significant increase in infected males (*p* ≤ 0.01; ANOVA), compared with non-infected males (Fig. 4D). The infected females showed the smallest proportion of small EVs, with a proportional increase in large EVs (Fig. 4D), albeit not significant when compared to the non-infected females. Analysis of positively labelled EVs by dSTORM imaging confirmed a mixture of pan-EV and TT positive EVs across all four sample groups (Fig. 4E), in addition to pan-EV positive (Fig. 4F) and TT positive labelled EVs (Fig. 4G) across all four groups. When comparing double-stained EVs (pan-EV and TT positive; Fig. 4E), a trend of increased EV numbers was observed in the infected male crabs compared to the non-infected male crabs, similar to what was observed for the total EV numbers identified by NTA (Fig. 4A). There was a significant difference observed in TT positive EVs comparing the female infected and non-infected crabs (*p* ≤ 0.05, *t*-test; Fig. 4G), which was reflected in increased pan-EV positive EVs between the two groups, albeit not statistically significant (Fig. 4F). This correlated with a shift in subpopulations of small and large EVs for the female crabs observed by NTA (Fig. 4D).

### EV protein cargoes and associated pathway enrichment comparing infected and non-infected male and female crabs

LC-MS/MS analysis was carried out for EV protein cargoes of the four study groups, infected and non-infected male and female crabs respectively, with shared and unique protein hits per group summarised in Table 1.

**Table 1:** Protein hits identified by LC-MS/MS in EV cargoes of non-infected and infected male and female crabs are presented. The protein hits are matched against the UniProt database for Heterotremata (https://www.uniprot.org/taxonomy/116706). A tick (v) indicates that the protein was identified in the respective group. Protein ID, Protein name,name of species hit, Mascot score and number of sequences (“No seq”) are shown.

Protein ID	Protein Name	Species hit name	Male non-inf	Male inf	Female non-inf	Female inf	Mascot Score¥	No seq.
A0A6J3WT23	Cryptocyanin	*Carcinus maenas*	v	v	v	v	538	8
A0AAW0UDB6	Cryptocyanin	*Scylla* *paramamosain*		v	v		1119	18
Q23707	Hemocyanin subunit 6	*Metacarcinus magister*			v	v	1208	19
Q5G2A4	Hemocyanin subunit 5	*Metacarcinus magister*	v	v	v	v	1497	23
Q5G2A5	Hemocyanin subunit 4	*Metacarcinus magister*	v	v	v	v	1392	22
Q5G2A6	Hemocyanin subunit 3	*Metacarcinus magister*	v	v	v	v	1276	20
A2I5Y6	Hemocyanin subunit 3	*Portunus pelagicus*			v	v	68	2
Q5G2A7	Hemocyanin subunit 2	*Metacarcinus magister*	v	v	v	v	1020	17
A0A8J4YCV9	Hemocyanin subunit 2	*Chionoecetes opilio*		v			611	11
Q5G2A8	Hemocyanin subunit 1	*Metacarcinus magister*	v	v	v	v	954	16
A0A0U1ZVT9	Hemocyanin subunit 1	*Scylla paramamosain*			v		355	6
A0A223G1C8	Hemocyanin subunit 1	*Scylla serrata*				v	385	7
A0A8J4YE88	Hemocyanin C chain	*Chionoecetes opilio*	v	v		v	705	10
A0A0N9EJJ5	Hemocyanin	*Eriphia* *verrucosa*	v	v	v	v	602	9
Q9NGL5	Hemocyanin subunit	*Callinectes sapidus*	v	v	v	v	630	9
P83175	Hemocyanin subunit B	*Cancer pagurus*			v	v	87	1
A0AAW0UEU5	Uncharacterized Protein	*Scylla paramamosain*	v			v	355	7
A0AAW0T4R9	Hemocyanin	*Scylla paramamosain*	v	v		v	314	4
A0A5B7DXY6	Actin, alpha cardiac muscle 2	*Portunus trituberculatus*	v		v	v	228	3
A0A8J4YGG3	Actin, alpha cardiac muscle 2	*Chionoecetes*		v			270	4
A0A8J4XRC7	Actin, muscle	*Chionoecetes opilio*		v	v	v	404	6
A0A8J5CJ27	Actin, muscle	*Chionoecetes opilio*		v			349	5
A0A0S4XQM5	C-type lectin	*Scylla paramamosain*	v	v	v	v	129	2
A0A0P4W8W5	Actin	*Scylla* *olivacea*	v	v			363	5
A0A482LUC2	Actin 1	*Cancer borealis*		v			416	7
A0A0P4WDB5	Actin	*Scylla* *olivacea*		v			336	4
A0A0P4WKL4	Histone H2B	*Scylla* *olivacea*	v				80	2
A0A8J5D0L6	RNA-directed DNA Polymerase	*Chionoecetes opilio*	v	v	v	v	59	2
A0A8J4YCL7	BUD13 homolog	*Chionoecetes opilio*	v	v		v	51	1
A0AAW0TXL9	Elongation factor G, mitochondrial	*Scylla paramamosain*	v		v		41	1
A0A8J4Y6W3	Failed axon Connections	*Chionoecetes opilio*	v	v	v	v	37	1
A0A8J4XRQ6	Piwi-like protein Siwi	*Chionoecetes opilio*	v				35	1
A0A8J5C0G6	Uncharacterized Protein	*Chionoecetes opilio*	v				35	1
A0AAW0T4M2	Tyrosinase copper-binding domain-containing protein	*Scylla paramamosain*		v			469	7
A0AAW0T4D3	Tyrosinase copper-binding domain-containing protein	*Scylla paramamosain*			v		400	7
A0AAW0T582	Tyrosinase copper-binding domain-containing protein	*Scylla paramamosain*				v	412	8
A0A0P4WKT2	Glyceraldehyde-3-phosphate dehydrogenase	*Scylla olivacea*		v		v	120	2
A0A976YI25	Arginine kinase Cal b 2.0101	*Callinectes bellicosus*		v	v		64	2
A0A5B7CMF4	Beta-1,3-glucan-binding protein	*Portunus trituberculatus*		v		v	35	1
A0AAW0UMV8	Alpha-2-macroglobulin-like	*Scylla paramamosain*		v			34	1
A0A5Q0TZF1	Vitellogenin	*Callinectes toxotes*			v	v	191	2
Q1L7 × 1	Vitellogenin	*Portunus* *trituberculatus*			v	v	150	2
A0A8J5CZJ5	Vitellogenin	*Chionoecetes opilio*				v	29	1
A0AAW0TWD4	Uncharacterized Protein	*Scylla paramamosain*			v		39	1
A0A5B7F1R2	Uncharacterized Protein	*Portunus trituberculatus*			v		38	1
A0A5B7HYW1	Uncharacterized Protein	*Portunus trituberculatus*			v		38	1
A0A0N7ZA62	Chorein N-terminal domain-containing protein	*Scylla olivacea*			v		35	1
A0A7T1ND56	Discoidal lipoprotein and beta-glucan binding protein	*Scylla paramamosain*				v	123	2
A0A0N7ZDP4	Large ribosomal subunit protein uL5	*Scylla olivacea*				v	36	1
A0A5B7FA28	Uncharacterized Protein	*Portunus trituberculatus*				v	34	1
A0A0P4W036	Enkurin domain-containing protein	*Scylla olivacea*				v	34	1

¥Ions score is −10*Log(P), where P is the probability that the observed match is a random event. Individual ions scores > 33 indicate identity or extensive homology(p < 0.05). Protein scores are derived from ions scores as a non-probabilistic basis for ranking protein hits.

**Fig. 5:**
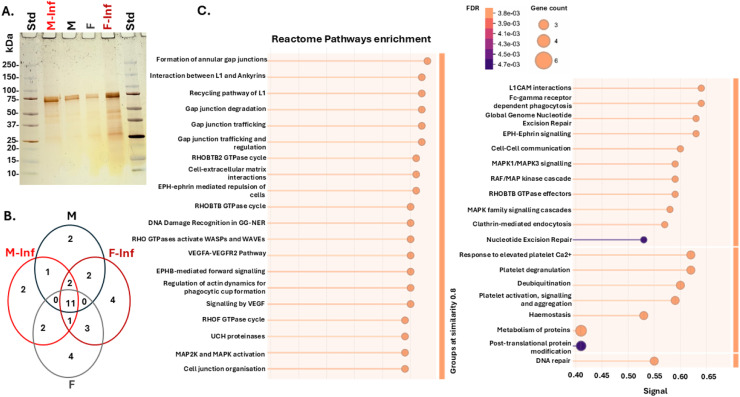
Crab haemolymph EV protein cargo analysis. A. A silver stained 5–20% TGX gel showing EV protein content for the four groups. B. Venn diagram showing shared and unique protein hits in EV cargoes (a total of 34 proteins were identified) of the four groups. **C.** Reactome Pathway enrichment analysis for total EV protein cargoes identified in the four experimental crab groups, highlights roles in cellular communication and immunity. FDR values are indicated alongside Gene count for the pathway enrichment analysis.

Analysis of the EV-associated proteins by SDS-PAGE (4–20% TGX gels) with silver-staining is shown in Fig. 5A, with numbers of shared and unique protein hits identified by LC-MS/MS summarised in the Venn diagram in Fig. 5B When assessing pathway enrichment for all identified protein hits in the crab EVs from the four groups in STRING, 15 proteins were identified for Crustacea, using the *Penaeus vannamei* database for further pathway enrichment analysis, as a crab specific database is not available in STRING. In total, 39 Reactome pathways, mainly relating to cell communication and immunity, were identified for the hits available in STRING and are presented in Fig. 5C, based on signal and false discovery rate (FDR). This included pathways involved in Gap junction formation, trafficking, regulation and degradation; Cell-extracellular matrix interactions; Cell-cell communication; DNA damage pathways and DNA repair; VEGF signalling pathways; Regulation of actin dynamics and phagocytic cup formation; Phagocytosis pathways; Clathrin mediated endocytosis; MAPK pathways; RHO GTPase pathways; L1CAM interactions; Platelet degranulation and response to elevated Ca^2+^; Haemostasis; Protein metabolism; and Post-translational protein modifications (Fig. 5C).

#### EV proteome cargoes only identified in infected male crabs

**Fig. 6:**
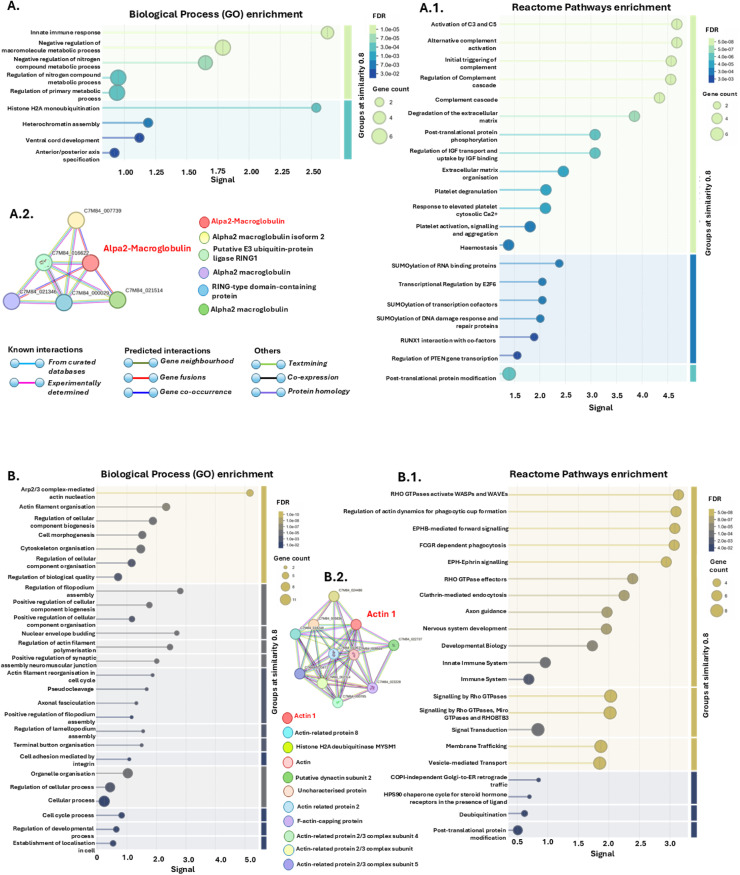
Unique EV proteome hits in infected male crabs. A. Alpha-2-macroglobulin associated pathway enrichment analysis for Biological GO pathways (A) and for Reactome pathways (A.1), while the PPI network analysis for Alpha-2-Macroglbulin is shown in A.2. **B.** Actin 1 associated pathway enrichment analysis for Biological GO pathways (B) and for Reactome pathways (B.1), while the PPI network analysis for Actin-1 is shown in B.2. Coloured nodes represent the query proteins and first shell of interactors; with protein names indicated in the legend. Coloured lines indicate whether protein interactions are identified via known interactions (curated databases, experimentally determined), predicted interactions (gene neighbourhood, gene fusion, gene co-occurrence), or via text mining, co-expression, or protein homology (see the colour key for connective lines included in the figure).

Two protein hits which were identified only in the EV proteome of the male infected group were Alpha-2-macroglobulin-like protein and Actin 1; these were separately assessed for protein-protein interactions and associated Biological Process Gene Ontology (GO) and Reactome pathway enrichment analyses (Fig. 6A-B).

Enriched Biological GO pathways associated with alpha-2-macroglobulin reflected its role in innate immune responses and regulation of primary metabolic processes, as well as associations with Histone H2A regulation, Heterochromatin assembly, Ventral cord development; and Anterior/posterior axis specification (Fig. 6A). Reactome pathway enrichment analysis highlighted roles for alpha-2-macroglobulin in Complement pathway activation and regulation; Platelet signalling (activation, degranulation, aggregation); Extracellular matrix organisation and degradation; Post-translational protein modifications (phosphorylation, SUMOylation), Insulin growth factor mediated pathways, Haemostasis, Transcriptional regulation; DNA damage and repair responses; RUNX1 pathways; and PTEN gene transcription (Fig. 6A.1). It must be highlighted that these interpretations are based on inferred pathways via STRING analysis using the *P. vannamei* STRING database.

Enriched Biological GO pathways associated with Actin 1 reflected its various roles in Cytoskeleton organisation; Filopodium assembly; Lamellipodium assembly; Cell morphogenesis; Cellular compartment regulation; Regulation of synaptic assembly at neuromuscular junction; Actin filament organisation in the cell cycle, Pseudocleavage; Terminal button organisation; Integrin mediated cell adhesion; Organelle organisation; Regulation of cellular processes and cell cycle processes; and Regulation of developmental processes (Fig. 6B). Reactome pathway enrichment analysis for Actin 1, furthermore highlighted roles in Immune and Innate immune system functions; Formation of actin dynamics for phagocytic cup formation and FCGR dependent phagocytosis; Clathrin-mediated endocytosis; RHO GTPase pathways; EPHB-mediated signalling; Axon guidance; Nervous system development; Developmental Biology; Signal transduction; Membrane trafficking; Vesicle-mediated transport; Golgi-to-ER retrograde traffic; and Post-translational protein modification (Fig. 6B1). It must be highlighted that these interpretations are based on inferred pathways via STRING analysis on the *P. vannamei* database.

#### EV proteome cargoes only identified in EVs of infected female crabs

**Fig. 7:**
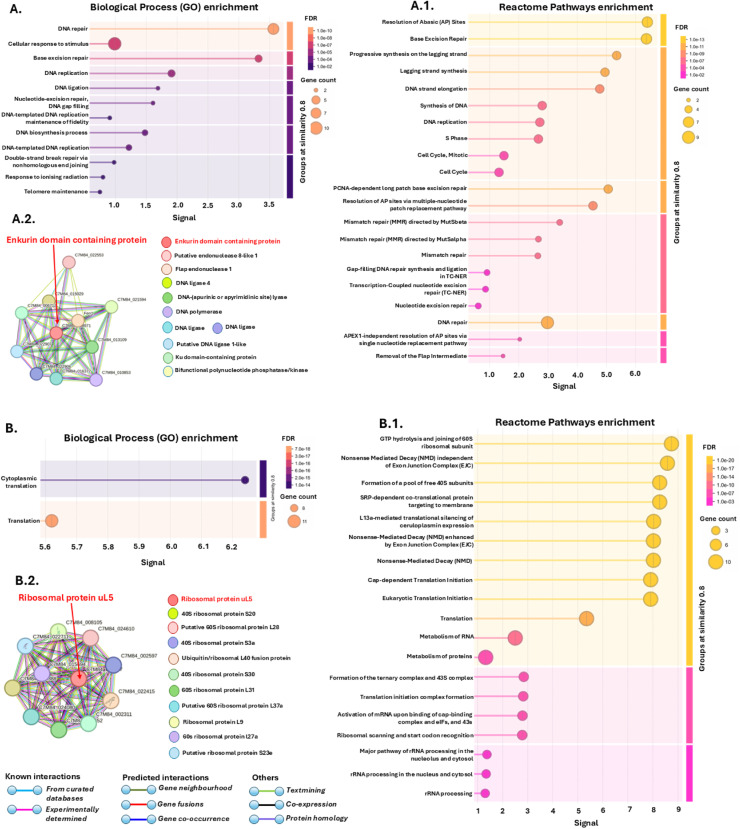
Protein-protein interaction and pathway enrichment analysis for two of the four unique hits for infected female crab EV proteomes: A. Ribosomal protein uL5; and **B.** Enkurin domain containing protein. A.1 and B.1: Enriched Biological GO and Reactome pathways are shown for both proteins, respectively. A.2 and B.2 show the PPI networks for Ribosomal protein uL5 (A.1) and Enkurin domain containing protein (B.1), respectively. The coloured nodes represent the query proteins and first shell of interactors; with protein names indicated in the legend. Coloured lines indicate whether protein interactions are identified via known interactions (curated databases, experimentally determined), predicted interactions (gene neighbourhood, gene fusion, gene co-occurrence), or via text mining, co-expression, or protein homology (see the colour key for connective lines included in the figure).

Four protein hits identified only in the EVs of the female infected groups were Discoidal lipoprotein and beta-glucan binding protein; Large ribosomal subunit protein uL5; Enkurin domain containing protein and an uncharacterized protein. Both ribosomal protein uL5 and Enkurin domain protein were found in the STRING *Penaeus vannamei* database, with protein-interaction networks and associated Biological GO and Reactome pathway analyses for both proteins shown in Figs. 7A-B.

Enriched Biological GO pathways associated with Enkurin domain containing protein showed roles in DNA biosynthesis, repair, replication and ligation; Cellular response to stimulus; Response to ionising radiation; and Telomere maintenance (Fig. 7A). Reactome pathway enrichment emphasised associations with DNA biosynthesis and repair pathways; Mismatch repair pathway; and regulation of the Cell cycle (Fig. 7A.1).

Enriched Biological GO pathways associated with Ribosomal protein uL5 highlighted roles in Cytoplasmic translation; and Translation (Fig. 7B). Reactome pathway enrichment highlighted roles in Ribosomal processing; Translation; rRNA processing; RNA metabolism; and Protein metabolism (Fig. 7B1). It must be highlighted that these interpretations are based on inferred pathways via STRING analysis, using the *P. vannamei* database.

## Discussion

This is the first study to report EVs in Atlantic rock crab and to assess EV signatures and associated EV proteomes in *C. irroratus* with shell disease. The pathology was recently characterised with associated histopathological analysis showing synergistic effects of various bacterial and oomycete pathogens [15]. The current study aimed to characterise EV signatures in *C. irroratus* haemolymph to identify some of EVs’ roles in cell communication and immunity and to assess these as putative infection biomarkers. Based on nanoparticle tracking analysis for size distribution, crab haemolymph EVs fell mainly within the 50–250 nm range (and overall range of 40–400 nm), with majority of the EVs in the 100–200 nm size range, which is similar to EV profiles reported in shore crab *C. maenas* [31] and mud crab *Scylla paramamosain* [38]. Positive detection of tetraspanin and flotillin-1 EV markers corresponds other studies on EVs in crustacea [[20], [31], [32]], using cross-reactive commercially available antibodies. In our current study, there was some observed increase of EV numbers in infected compared with non-infected male crabs, which was mirrored by a significant increase in medium sized EVs in the infected crabs, and some increase in co-labelled PanEV+ and TT+ EVs by single molecule detection, using dSTORM imaging. In the female infected crabs, some shift to larger EV profiles was observed, albeit not significant, and this coincided with a decrease in small EVs, which correlated with a significant reduction of TT positive EVs by dSTORM imaging in the infected female crabs. The direct relevance of such EV subpopulation changes in response to infection may be of considerable interest and will require further investigation in larger sample sizes in future studies. It may furthermore be postulated whether EV mediated responses vary depending on type of infection, as well as in different crustacean species. For example, a reduction in EV numbers was observed in haemolymph of shore crabs (*C. maenas*) infected with *Hematodinium* sp., with more marked differences in males than females [31]. In Chinese mitten crab (*Eriocheir sinensis*), small EVs (exosomes) were identified to have critical roles in inhibiting *Spiroplasma eriocheiris* infection via modulation of phagocytosis [33]. In mud crab (*Scylla paramamosain*) challenged with white spot syndrome virus (WSSV), exosomes (small EVs) were found to mediate apoptosis pathways as part of the innate immune response [38].

When assessing the *C. irroratus* haemolymph EV proteomes by LC-MS/MS in conjunction with STRING pathway analysis in all four study groups, our findings highlighted roles in pathways associated with a range of cell-communication and immune mechanisms, with some sex-specific differences observed. Shared EV associated proteins in both infected and non-infected groups included Hemocyanin, C-type lectin, Actin, RNA-directed DNA polymerase, Failed axon Connections, Tyrosinase copper-binding domain-containing protein, BUD13 homolog, Elongation factor G, and Mitochondrial Arginine kinase. Some additional shared proteins in the infected crabs included Beta-1,3-glucan-binding protein and Glyceraldehyde-3-phosphate dehydrogenase. When assessing sex differences in infected crabs, two protein hits which were only associated with male crab EVs were actin-1 and alpha-M-macroglobulin, while three protein hits specific to infected female crab EVs were Discoidal lipoprotein and beta-glucan binding protein, Enkurin domain containing protein, and ribosomal protein uL5. The individual proteins are briefly discussed below regarding their known biological roles and in association with the crustacea literature.

### Shared EV associated protein cargoes in male and female crabs

The following EV cargoes were identified in all four study groups:

**Hemocyanin** (subunits 1, 2, 3, 4 and 5) has highly conserved copper-oxygen-binding sites, reversibly binds oxygen and has key roles in supplying tissues with oxygen [39,40]. Subunits 1, 2 and 3 are constitutive, but subunits 4, 5 and 6 are upregulated during development and progressively recruited, with associated changes in oxygen affinity [41]. Haemocyanin subunit 6 and B were in addition identified in the EVs of the female crabs (infected and non-infected), possibly indicating differences in oxygen binding potential between the groups. Antibacterial and phagocytic activity of hemocyanin has been reported in red swamp crayfish (*Procambarus clarkii*) [42]. It must be noted that crustacean haemolymph contains high levels of hemocyanin, and this may have contributed to the high score observed for this protein hit in the proteomic analysis, as it cannot be fully excluded that a possible co-sediment may have been captured with the EV isolation.

**Cryptocyanin** is closely related to hemocyanin but lacks the ability to bind oxygen. It has been identified to have crucial roles in the formation of new exoskeleton and involvement in regulation of molting hormones [39]. A study comparing male and female mud crabs (*Scylla olivacea*) reported significantly lower abundance of cryptocyanin protein in female crabs compared with male crabs during the intermolt stage [43].

**C-type lectins** are important Ca²⁺-dependent pattern recognition receptors in the immune system, with roles in development and host defence in vertebrates and invertebrates and have been reported in various crab species. For example, in Chinese mitten crab (*Eriocheir sinensis*) binding to a range of PAMPs and microorganisms have been reported [44]. In swimming crab (*Portunus trituberculatus*) roles in agglutination, pathogen recognition, opsonisation and phagocytosis are reported [[45], [46], [47]]. Roles for C-lectins in bacterial agglutination activity and encapsulation activity of hemocytes are also described in mud crab (*Scylla paramamosain*) [48]. Roles in the intestine-hemocyte axis in regulating antibacterial immunity in *E. sinensis* have also been identified [49].

**Actin, alpha cardiac muscle 2** is part of the actin isoform family, which plays multifaceted roles in cellular processes including cytoskeletal organisation and muscle function [50]. Various skeletal muscle, heart and cytoplasmic types of actins were reported in lobster (*Homarus americanus*) [51]. In crustacea, actins play various roles in intracellular and extracellular functions, including in connective tissue and the integument [52].

**RNA-directed DNA polymerases** have key roles in the eukaryotic transcription apparatus [53]. In a linkage mapping study of swimming crab (*Portunus trituberculatus*), growth-related roles were suggested [54]. Possible roles in organismic response to environmental change have also been explored in boreal spider crab (*Hyas araneus*) [55].

**Failed axon Connections (FAXC)** has been described in *Drosophila*, with roles in the development of axon bundle in the central nervous system [56]. FAXC has been identified to interact with mitochondria pathways and have roles in tumour development and cell proliferation [57,58]. In crab, no specific roles have been reported to date.

In addition, **Tyrosinase copper-binding domain-containing protein** was identified in EVs of all groups except non-infected males. They associate with hemocyanins. Copper binding proteins have been reported in Chinese mitten crab (*Eriocheir sinensis*), with elevated levels in haemocytes post *Vibrio anguillarum* challenge and involvement in acute immune responses [59]. Roles in the shell hardening process during the molt cycle have also been identified [60]; in the current study all crabs included were inter-moult (hard shell).

**BUD13 homolog** was identified in EVs of all groups except non-infected females. It is a highly conserved RNA-binding protein with roles in the spliceosome complex by pre-mRNA splicing [61]. BUD13 has roles in metabolic regulation [62] and identified to be upregulated in lymphoma [63]. In the mud crab (*Scylla paramamosain*) it has been identified to be involved in osmotic regulation, with upregulation in response to a stressor of a sudden salinity drop [64].

**Elongation factor G, mitochondrial** was identified in EVs of non-infected males and females. It is phylogenetically conserved and has critical roles in protein synthesis within mitochondria [[65], [66], [67]]. It has roles in regulating normal mitochondrial function and is associated with mitochondrial disfunction and a range of oxidative phosphorylation disorders [68,69].

**Arginine kinase Cal b 2.0101** was identified in EVs of infected males and non-infected females. Arginine kinase is identified as a major pan-allergen in crustaceans, which induces IgE-mediated immune responses in humans [[70], [71], [72]]. It is similar to creatine kinase in vertebrates, and has key roles in cell metabolism, with roles in cellular energy metabolism in invertebrates [[73], [74]]. Arginine kinase was identified as up-regulated in gills of *Penaeus vannamei* infected with yellow head virus [75].

The following two proteins were shared in infected male and female crab EVs:

**Beta-1,3-glucan-binding protein**, is a main lipid transporter in crustacea and is also a key pattern recognition protein with important roles in immune defences [76]. It triggers innate immune responses by detecting beta-glucan and has antioxidant functions and aids clearance of pathogens. It has been identified to stimulate the prophenoloxidase activating system in Chinese mitten crab (*Eriocheir sinensis*) and rice field crab (*P. hydrodromus*) [77,78]. Given its role in innate defence against invading pathogens, its detection in both male and female infected crab haemolymph EVs aligns with EV cargo mediated transport as part of the immune response.

**Glyceraldehyde-3-phosphate dehydrogenase** plays multifaceted roles in metabolism, DNA repair, apoptosis and nuclear transport. In Chinese mitten crab (*Eriocheir sinensis*), it was identified as downregulated in response to cadmium exposure, highlighting roles in toxicity and oxidative stress responses [79]. Roles in immune responses in viral infection in Pacific white shrimp (*Litopenaeus vannamei*) infected with Infectious hypodermal and hematopoietic necrosis virus (IHHNV) have also been reported [80].

### Sex-specific differences identified for the EV proteome cargo

Some sex-specific differences were observed for EV protein cargoes. Two protein hits were only identified in non-infected male crab EVs:

**Histone H2B**, forms part of the histone octamer which is crucial for DNA packaging and chromatin stability. H2B variants are associated with changes in transcriptional activation and affect chromatin dynamics. H2B has been studied in relation to spermatogenesis in Chinese mitten crab (*Eriocheir sinensis*) [81], with crucial roles identified for chromatin decondensation in spermatozoa in several decapod species [82]. Histones, including H2B, are also involved in antimicrobial activity and are part of crustacea innate immune defence [[83], [84], [85]].

**Piwi-like protein Siwi** is part of the Piwi-interacting RNA pathway, which has been identified to be involved in antiviral defences [86].

One protein hit was identified only in EVs of the non-infected females:

**Chorein N-terminal domain-containing protein**, which is an evolutionary conserved domain present in a range of proteins with roles in lipid transport, endocytosis and autophagy trafficking pathways [87].

With respect to sex-specific cargoes shared between infected and non-infected individuals, **Actin** was identified for the male crabs. Actin plays multifaceted roles in cell-cell communication, intracellular motility and crucial roles in innate immunity including to environmental stressors [88,89].

Three protein hits were shared for the EVs of the non-infected and infected female groups (and not identified in the other groups), and these were:

**Hemocyanin subunit 6 and subunit B**, already discussed above, and may possibly reflect some differences in oxygen binding potential and immune responses between male and female crabs [42,41].

**Vitellogenin** was furthermore specific to the female crab EVs, which correlates with vitellogenin’s role as a precursor of yolk proteins. Vitellogenin has also been identified as a multivalent pattern recognition receptor, with important roles in innate immune responses, and binding capabilities for virions, glucan, peptidoglycans and lipopolysaccharides [90]. In Chinese mitten crab (*Eriocheir sinensis*) accumulation of Vitellogenin in the haemolymph plays critical roles in antimicrobial function in response to bacterial challenge [91].

The function of the proteins discussed above was reflected in enrichment identified in numerous GO Biological Pathways and Reactome pathways as reported in Fig. 5. This included pathways involved in Gap junction formation, trafficking, regulation and degradation; Cell-extracellular matrix interactions; Cell-cell communication; DNA damage pathways and DNA repair; VEGF signalling pathways; Regulation of actin dynamics and phagocytic cup formation; Phagocytosis pathways; Clathrin mediated endocytosis; MAPK pathways; RHO GTPase pathways; L1CAM interactions; Platelet degranulation and response to elevated Ca^2+^; Haemostasis; Protein metabolism; and Post-translational protein modifications.

#### Proteins only associated with infected male or female crab EVs, respectively

Proteins only identified in infected male crab EVs were Actin-1 and Alpha-2-macroglobulin.

**Actin-1** has multifaceted roles in immunity and cell communication due to its function as a structural constituent of the cytoskeleton, involvement in cell motility and ATP binding [[89], [92]]. This related to the identified enriched Biological GO pathways, which associated with Actin-1 reflecting its various roles in Cytoskeleton organisation including Filopodium and lamellipodium assembly; Cell morphogenesis, Integrin mediated cell adhesion; and Regulation of developmental processes (Fig. 6). Reactome pathway enrichment analysis for Actin 1, furthermore highlighted roles in Immune and Innate immune system functions (Fig. 6).

**Alpha-2-macroglobulin** has key innate immune functions and regulation metabolic processes [93]. Pathway enrichment analysis provided in Fig. 6 further identified the various associations with enriched Biological GO pathways including innate immune responses and regulation of primary metabolic processes, as well as associations with Histone H2A regulation, Reactome pathway enrichment analysis highlighted roles for alpha-2-macroglobulin in Complement pathway activation and regulation; Extracellular matrix organisation Transcriptional regulation; DNA damage and repair responses.

Proteins only identified in infected female crab EVs were Discoidal lipoprotein and beta-glucan binding protein, Large ribosomal subunit protein uL5 and Enkurin domain-containing protein.

**Discoidal lipoprotein and beta-glucan binding protein**, has been reported to have possible roles in lipid transport, but otherwise its roles are hitherto unidentified [76,94]. Its presence in relation to EVs of the infected group only may indicate roles in immunity and responses to shell disease. As this protein was not found in the STRING database, further pathway enrichment analysis could not be performed.

**Large ribosomal subunit protein uL5,** has key roles in ribosomal processing, translation and transcription [95]. Enriched Biological GO pathways highlighted roles in Cytoplasmic translation and Translation. Reactome pathway enrichment highlighted roles in Ribosomal processingRNA metabolism and Protein metabolism (Fig. 7).

**Enkurin domain-containing protein** is involved in cell migration pathways [96] and is a regulator in innate immune responses and host defence against intracellular pathogens [97]. Enriched Biological GO pathways associated with Enkurin domain containing protein showed roles in DNA repairand Telomere maintenance. Reactome pathway enrichment emphasised associations with DNA biosynthesis and repair pathwaysand regulation of the Cell cycle (Fig. 7).

Some study limitations need to be acknowledged. EV quantification by NTA for EV numbers was carried out on 10 females per group, but 20 males per group, which may have some influence on statistical analysis. A skewed sex ratio in sampling has been consistently observed for *C.irroratus* [[15], [98]], and this also affected our current study. Further comparisons on haemolymph EV concentration between groups should be carried out in larger sample sizes to confirm whether the observed sex-specific differences are consistent. For the proteomic EV cargo profiling, a pool of 10 samples per group was used, which may mask individual variability. Therefore, while some sex-specific differences were observed in the proteome analysis this may need some caution for interpretation and further validation in larger cohorts, including with respect to individual variability. While the current study relied on commercially available reagents from the EV Profiler 2 kit (ONI) and therefore on cross-reactivity for the EV capture and detection antibodies between species, EV labelling was clear both for the tetraspanin trio TT marker and the Pan-EV marker. Here, the crab EVs were bound to the chip with phosphatidylserine (PS) capture and positive staining of the EV markers was compared with positive human-derived control EVs supplied with the kit. EV specific antibodies, including CD63 and Flot-1 have been shown to cross react with a wide range of species, including other crustacea [31,32], and EV related tetraspanins are conserved in crustacea ([32]; (UniProt ROT68825.1; XP_063849688.1; ROT72465.1). Therefore, while the antibodies used were not crab-specific and some caution in interpretation is necessary, the data did show clear EV labelling detection of *C,irroratus* haemolymph EVs. Furthermore, while pathway enrichment analysis may provide valuable insights into EV mediated communication in *C. irroratus* via transport of protein cargoes, interpretations do need some caution given the limited literature on *C.irroratus* and the use of inferred pathways based on a representative crustacean species.

Changes in EV mediated cellular communication and associated proteome cargo changes have not been reported in relation to shell disease in crabs before and pose as new biomarkers for disease monitoring. Previous studies in different crab species have indicated roles for EVs in *Haemotidium* sp. infection [31], WSSV infection [38] and *S. eriocheiris* infection [33]. In addition to studying EV-mediated responses to infection *per se*, changes in environmental conditions, including temperature and pollution, are also important synergistic factors affecting marine animal health. There is growing evidence that EVs may be useful biomarkers to reflect such changes. This has for example been shown for some fish species in stressed conditions due to water temperature change [[28], [99]]. Expanding EV research in wildlife and aquaculture is a large field that remains relatively unexplored with a vast scope for establishing biomarkers. To our knowledge, this is the first study to use dSTORM imaging for crustacea EVs, which opens a platform for further detailed studies tracking specific EV cargoes (including proteins and RNA species) to EV subtypes in response to pathogenic infection and other immune-challenges, including environmental temperature changes and pollutants.

## Conclusion

This study provides the first analysis of Atlantic rock crab (*C.irroratus)* haemolymph EV signatures in response to shell disease, assessing female and male crabs, with some sex-differences observed. EV numbers were somewhat elevated in haemolymph of the infected male crabs with shell disease, compared with unaffected males. Changes in EV subpopulations were also observed. EV protein cargoes of both male and female crabs were modified, as identified by SDS-PAGE and silver-staining, and upon proteomic analysis more protein hits were identified for the female infected group. Overall, crab haemolymph EV protein cargoes were associated with various cell-communication and immune pathways. Two protein EV cargoes identified only in infected male crabs were alpha-2-Macroglobulin, which has key innate immune functions, and actin-1 which has multifaceted roles in immunity and cell communication. Three specific EV protein cargoes identified in EVs of the infected female crabs were Enkurin domain containing protein, which has roles in DNA repair and the cell cycle; ribosomal protein Lu5, which has roles in translation, metabolism and RNA processing; and Discoidal lipoprotein and beta-glucan binding protein with unidentified roles. While our study supports the potential of haemolymph EVs as disease-related indicators, their use as practical biomarkers will require further validation in larger cohorts, with assessment for individual variations, also in context with disease-stage and additional comparison with other environmental or infectious stressors. The presented findings pave way for using haemolymph EVs as biomarkers in marine shellfish for disease monitoring and assessment of environmental changes.

## Funding

The study was in part funded by UIRCS and SINRC, and supported by UKRI funding through access to STFC's Central Laser Facility's Octopus Facility Harwell Science and Innovation Campus (LSF 26130010).

## CRediT authorship contribution statement

**Sigrun Lange:** Writing – review & editing, Writing – original draft, Visualization, Validation, Supervision, Resources, Project administration, Methodology, Investigation, Funding acquisition, Formal analysis, Data curation, Conceptualization. **Sindri Gíslason:** Writing – review & editing, Resources, Methodology, Investigation, Data curation. **Sarah R. Needham:** Writing – review & editing, Resources, Methodology, Data curation. **Benjamin M. Davis:** Writing – review & editing, Methodology, Formal analysis, Data curation. **Igor Kraev:** Visualization, Resources, Methodology. **Hermann Dreki Guls:** Methodology. **Sandra Dögg Georgsdóttir:** Methodology. **Árni Kristmundsson:** Writing – review & editing, Visualization, Resources, Methodology, Investigation. **Halldór Pálmar Halldórsson:** Writing – review & editing, Validation, Supervision, Project administration, Investigation, Funding acquisition, Data curation.

## Declaration of competing interest

The authors declare that they have no known competing financial interests or personal relationships that could have appeared to influence the work reported in this paper.

## Data Availability

Data will be made available on request.

## Appendix Supplementary materials

Image, application 1
