# Supplementary material for: Extracellular vesicle signatures are modified in haemolymph of atlantic rock crab (Cancer irroratus) infected with shell disease – a Study from Icelandic waters
Source: Comp Immunol Rep. 2026 Jul 9;11:200299. doi: 10.1016/j.cirep.2026.200299 (PMC13382624; doi:10.1016/j.cirep.2026.200299)
Supplement: Supplementary file 1 [file mmc1.docx]

**Supplementary Table 1.** Rock crabs sampled for the study. Only individuals in the inter-molt stage (hard shell) were selected to ensure consistency in heamolymph protein concentrations. Sex is indicated as male or female (M/F), carpace width (CW) is indicated in cm, and number of black spots (lesions) indicative of shell disease are listed for Claws (C), Dorsal carapace (D), Edge of carapace (E ) and Legs (L). Non-infected (control) crabs had no black spots/lesions. The study utilised 20 infected males, 20 non-infected males, 10 infected females and 10 non-infected females.

| **Sex** | **CW** | **Black spots** | | | |  |
| --- | --- | --- | --- | --- | --- | --- |
| (M/F) | cm | C | D | E | L |  |
|  |  |  |  |  |  | **Shell Disease / Control** |
| M | 12.6 | 0 | 2 | 1 |  | Shell Disease |
| M | 13.7 |  | 3 | 2 |  | Shell Disease |
| M | 13.7 |  | 3 | 2 |  | Shell Disease |
| M | 13.1 | 1 | 3 | 2 | 1 | Shell Disease |
| M | 13.8 | 2 | 3 | 2 | 1 | Shell Disease |
| M | 12.3 | 2 | 2 | 2 |  | Shell Disease |
| M | 13.7 | 2 | 3 | 2 | 1 | Shell Disease |
| M | 13.7 | 2 | 2 | 2 |  | Shell Disease |
| M | 13 | 2 | 3 | 2 |  | Shell Disease |
| M | 13.7 | 1 | 2 | 1 |  | Shell Disease |
| M | 12.3 | 2 | 3 | 2 |  | Shell Disease |
| M | 13.7 |  | 3 | 2 |  | Shell Disease |
| M | 13.2 | 2 | 2 | 2 |  | Shell Disease |
| M | 12 | 0 | 3 | 2 |  | Shell Disease |
| M | 12.4 |  | 3 | 2 |  | Shell Disease |
| M | 12.2 | 2 | 2 | 1 | 1 | Shell Disease |
| M | 13.1 | 2 | 3 | 2 |  | Shell Disease |
| M | 12.2 | 1 | 2 | 2 | 1 | Shell Disease |
| M | 13.3 | 0 | 2 | 2 | 1 | Shell Disease |
| M | 13.5 | 1 | 3 | 2 |  | Shell Disease |
| M | 11.5 |  |  |  |  | Control |
| M | 11.6 |  |  |  |  | Control |
| M | 11.7 |  |  |  |  | Control |
| M | 11.8 |  |  |  |  | Control |
| M | 12.3 |  |  |  |  | Control |
| M | 11.7 |  |  |  |  | Control |
| M | 11.8 |  |  |  |  | Control |
| M | 10.4 |  |  |  |  | Control |
| M | 11.9 |  |  |  |  | Control |
| M | 12.5 |  |  |  |  | Control |
| M | 12.7 |  |  |  |  | Control |
| M | 11.6 |  |  |  |  | Control |
| M | 12.4 |  |  |  |  | Control |
| M | 11.3 |  |  |  |  | Control |
| M | 11.5 |  |  |  |  | Control |
| M | 11.5 |  |  |  |  | Control |
| M | 12 |  |  |  |  | Control |
| M | 11.2 |  |  |  |  | Control |
| M | 11.2 |  |  |  |  | Control |
| M | 11.7 |  |  |  |  | Control |
| F | 9.2 | 2 | 1 | 2 |  | Shell Disease |
| F | 8.9 | 2 | 2 | 2 | 1 | Shell Disease |
| F | 8.8 | 2 | 2 | 2 |  | Shell Disease |
| F | 9 | 2 | 2 | 2 | 1 | Shell Disease |
| F | 9.3 | 2 | 2 |  |  | Shell Disease |
| F | 9.5 | 2 | 1 | 1 | 1 | Shell Disease |
| F | 9.4 | 1 | 1 | 1 |  | Shell Disease |
| F | 8.2 |  | 1 | 1 | 1 | Shell Disease |
| F | 8.5 | 0 | 1 | 1 |  | Shell Disease |
| F | 8.4 |  |  | 1 |  | Shell Disease |
| F | 8.6 |  |  |  |  | Control |
| F | 8.7 |  |  |  |  | Control |
| F | 8.4 |  |  |  |  | Control |
| F | 8.4 |  |  |  |  | Control |
| F | 8 |  |  |  |  | Control |
| F | 7.8 |  |  |  |  | Control |
| F | 8.2 |  |  |  |  | Control |
| F | 7.6 |  |  |  |  | Control |
| F | 8 |  |  |  |  | Control |
| F | 8.5 |  |  |  |  | Control |
